# Integral Kinetic Model for Studying Quercetin Degradation and Oxidation as Affected by Cholesterol During Heating

**DOI:** 10.3390/ijms11082805

**Published:** 2010-07-29

**Authors:** John-Tung Chien, Da-Jung Hsu, Baskaran Stephen Inbaraj, Bing-Huei Chen

**Affiliations:** Department of Food Science, Fu Jen University, Taipei 242, Taiwan; E-Mails: 002604@mail.fju.edu.tw (J.T.C.); sinbaraj@yahoo.com (B.S.I.)

**Keywords:** quercetin, cholesterol oxidation, kinetic study

## Abstract

The degradation and oxidation of quercetin, as affected by cholesterol during heating at 150 °C, was kinetically studied using non-linear regression models. Both TLC and HPLC were used to monitor the changes of quercetin, cholesterol and cholesterol oxidation products (COPs) during heating. The formation of COPs, including triol, 7-keto, 7α-OH and 7β-OH, was completely inhibited during the initial 30 minute heating period in the presence of 0.02% quercetin, accompanied by reduction in cholesterol peroxidation and degradation. However, the quercetin degradation or oxidation proceeded fast, with the rate constants (h^−1^) in the presence of nitrogen, oxygen and the combination of oxygen and cholesterol being 0.253, 0.868 and 7.17, respectively. When cholesterol and quercetin were heated together, the rate constants (h^−1^) of cholesterol peroxidation, epoxidation and degradation were 1.8 × 10^−4^, 0.016 and 0.19, respectively. The correlation coefficients (r^2^) for all the oxidative and degradation reactions ranged from 0.82–0.99. The kinetic models developed in this study may be used to predict the degradation and oxidation of quercetin as affected by cholesterol during heating.

## 1. Introduction

Flavonoids are ubiquitously distributed in food plants with approximately 90% occurring as glycosides in nature [1,2]. Among flavonoids, quercetin and its glycoside derivatives are the most abundant in plant vegetables [2,3], which have been reported to be effective in scavenging free radicals during lipid oxidation [4–6]. In addition, several studies have demonstrated that quercetin is more effective than α-tocopherol and BHT in preventing cholesterol oxidation even at a low level [7–9].

To date, the kinetic study of the cholesterol oxidation pathway during heating has been well documented, showing the cholesterol oxidation is initiated by the second-order free radical chain reaction to form cholesterol hydroperoxides, followed by degradation to generate various cholesterol oxidation products (COPs), including 5,6α-EP, 5,6β-EP, 7α-OH, 7β-OH, 7-keto and triol [10–13]. In a recent study, Xu *et al*. [14] have shown the presence of four fatty acids, namely, stearic acid, oleic acid, linoleic acid and α-linolenic acid, to promote oxidation of both cholesterol and β-sitosterol. As the consumption of COPs in excess may be detrimental to human health [15,16], the inhibition of COPs formed during heating of cholesterol-rich foods is of great importance.

Up until now, there is a lack of data regarding the inhibition mechanism of cholesterol peroxidation as affected by quercetin. In a previous study dealing with development of a kinetic model for studying the effect of quercetin on cholesterol oxidation during heating, the reaction rate constants (h^−1^) for epoxidation, dehydration and dehydrogenation of COPs, as well as free radical chain reaction and thermal degradation of cholesterol were reduced greatly after incorporation of quercetin [17]. However, the degradation or oxidation of quercetin remained unknown. In this report, our objective was to develop an integral mathematic model for studying the thermal degradation or oxidation of quercetin in the presence and absence of cholesterol. Additionally, the cholesterol peroxidation, epoxidation and degradation as affected by quercetin were determined. The result of this kinetic study may provide a further insight in elucidating the mechanism of quercetin degradation and oxidation as affected by cholesterol during heating.

## 2. Results and Discussion

### 2.1. Changes of Quercetin during Heating with and without Cholesterol

**Figure 1:**
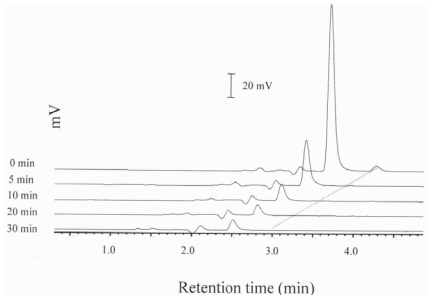
HPLC chromatograms of quercetin during heating at 150 °C under combination of oxygen and cholesterol.

As an antioxidant, quercetin was reported to be more effective than α-tocopherol in inhibiting lipid hydroperoxide formation even at a low level [7,8]. Of the various quercetin levels (0.02, 0.01, 0.005 and 0.002% of 100 mg cholesterol), we found that with the exception of 0.02% quercetin, for all other levels the quercetin were drastically degraded during the initial 10-min heating at 150 °C in the presence of cholesterol. Additionally, a 0.02% level of quercetin was efficient in inhibiting most COPs formation, with a significant residual amount of quercetin over a 30-min heating period (Figure 1). Thus, a level of 0.02% quercetin was selected in our study to elucidate the mechanism involved during heating of quercetin in the presence and absence of cholesterol. A heating temperature of 150 °C was chosen as cholesterol is prone to undergo autoxidation in liquid form when the temperature reaches its melting point of 148.5 °C or higher [17]. Furthermore, thermal degradation of cholesterol has been reported to dominate over autoxidation at 200–300 °C [17]. It is also the temperature similar to that used for low-temperature frying of foods [17]. Thus, the heating temperature of 150 °C was selected in this study to reduce the loss of cholesterol due to thermal degradation and concomitantly accelerate the formation of COPs as well as to mimic the low-temperature frying conditions of foods.

**Figure 2:**
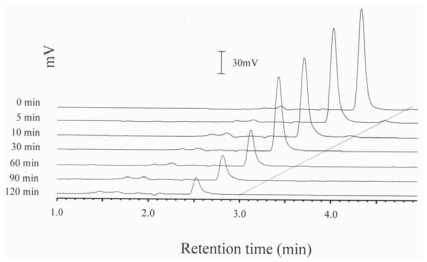
HPLC chromatograms of quercetin during heating at 150 °C under oxygen.

**Figure 3:**
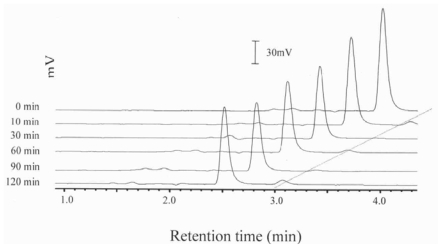
HPLC chromatograms of quercetin during heating at 150 °C under nitrogen.

**Figure 4:**
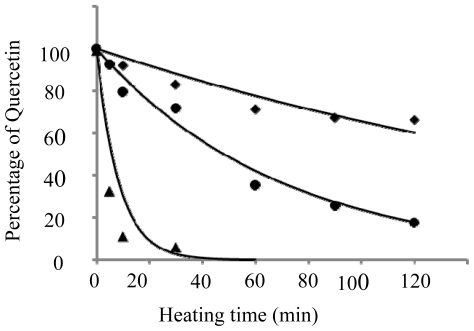
Changes of quercetin during heating at 150 °C. Quercetin was heated under nitrogen (◆), oxygen (●) or combination of oxygen and cholesterol (▴); the best fitting line (—). A concentration of 200 μg/g quercetin was used. Heating time (min)

**Figure 5:**
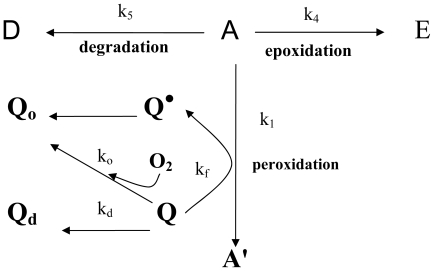
The possible pathways for quercetin degradation and oxidation as well as cholesterol peroxidation during heating at 150 °C. Cholesterol (A); 7-hydroperoxycholesterol (A′); 5,6-epoxycholesterol (E); degraded products (D); quercetin (Q); degraded plus oxidized product of quercetin (Q_d_, Q_o_); quercetin free radical (Q^•^). k_1_, k_4_, k_5_, k_o_, k_d_, and k_f_ are the corresponding rate constants.

Figures 1, 2 and 3 show the HPLC chromatograms of quercetin during heating at 150 °C in the presence of both oxygen and cholesterol, oxygen and nitrogen, respectively. With nitrogen, the amount of quercetin decreased gradually and a loss of 7.9, 17.2, 28.6 and 33.9% was generated after 10 min, 0.5, 1 and 2 h of heating, respectively (Figures 3 and 4). However, a greater loss of 20.4, 28.2, 64.8 and 82.4% of quercetin occurred when heated with oxygen for the same period (Figures 2 and 4). Apparently the quercetin degradation or oxidation exhibited a similar pattern when heated under nitrogen or oxygen, which is in accordance with a report by Makris and Rossiter [5,6]. In contrast, when heated with both cholesterol and oxygen, the level of quercetin drastically decreased to 10.9 and 5.9% after 10 and 30 min of heating, respectively (Figure 4). This is probably because of fast formation of cholesterol hydroperoxide through hydrogen abstraction at C-7 of cholesterol during the initial heating period [10,12], which may in turn accelerate quercetin degradation at 150 °C. As a result, the free radical involved in reactions of cholesterol oxidation and the quercetin degradation can be combined into a pathway as depicted in Figure 5. The major reaction pathways and rate constants for cholesterol peroxidation and quercetin degradation are shown in Tables 3 and 4. Comparatively, quercetin was degraded faster with cholesterol than without cholesterol, which should be caused by formation of free radicals at C-7 of cholesterol as indicated above. Additionally, quercetin may undergo oxidative degradation (Q→Q_o_) or non-oxidative degradation (Q→Q_d_) during heating at 150 °C, with the rate constant (h^−1^) being higher for the former than for the latter. The reaction pathways for peroxidation and epoxidation of cholesterol were the same as those described in a previous study [17], with the exception that some other COPs, including 7-OH, 7-keto, triol and cholestan-3β, 5α, 6-one, were excluded for kinetic study as they were not formed in the presence of quercetin over a heating period of 30 min.

### 2.2. Peroxidation and Epoxidation of Cholesterol during Heating

**Figure 6:**
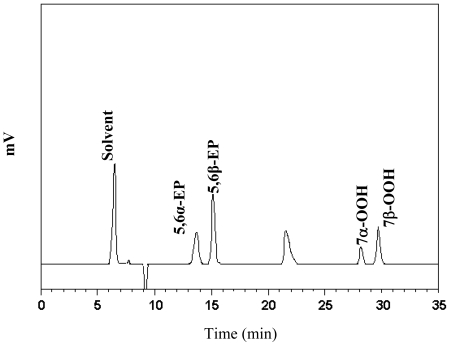
HPLC chromatogram of the acetone extract obtained from TLC after heating cholesterol with quercetin for 30 min.

Due to absence of cholesterol hydroperoxide standards, both 7α-OOH and 7β-OOH should be identified and quantified with care. According to Smith and Hill [18], both 7α-OOH and 7β-OOH could turn red after spraying *N*,*N*-dimethyl-*p*-phenylenediamine dihydrochloride onto a TLC plate. Both 7α-OOH and 7β-OOH were further subjected to HPLC analysis for identification. Figure 6 shows the HPLC chromatogram of the acetone extract of the red spots collected from TLC after heating cholesterol with quercetin for 30 min. A total of 5 peaks were present, in which 5,6α-EP, 5,6β-EP, 7α-OOH and 7β-OOH were identified. Both 7α-OOH and 7β-OOH were tentatively identified based on the retention behavior as described by Chien *et al*. [17].

**Figure 7:**
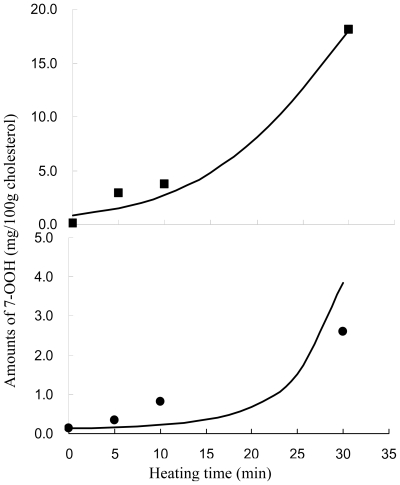
The amounts of 7-OOH (7α-OOH plus 7β-OOH) formed during heating of cholesterol with and without quercetin at 150 °C. Means of experimental data of cholesterol in the absence of quercetin (■), and cholesterol in the presence of quercetin (●); the best fitting line (—).

A total of eight major COPs, namely, 5,6α-EP, 5,6β-EP, 7-keto, 7α-OH, 7β-OH, 7α-OOH, 7β-OOH, and triol, were adequately resolved within 30 min by using the HPLC condition shown in the method section (Figure not shown). Interestingly, with quercetin, 7α-OH, 7β-OH, 7-keto and triol were undetected over a 30-min heating period (Figure 6). Conversely, without quercetin, all the eight COPs (5,6α-EP, 5,6β-EP, 7-keto, 7α-OH, 7β-OH, 7α-OOH, and 7β-OOH and triol) were formed gradually during heating, with the level of 7-OOH (7α-OOH plus 7β-OOH) being 0.82 mg (based on 100 g cholesterol) in the beginning, raised to 3.7 mg in 10 min and 18 mg in 30 min, whereas in the presence of quercetin, the initial level of 7-OOH was 0.13 mg, rose to 0.82 mg in 10 min and 2.6 mg in 30 min (Figure 7). Additionally, a small amount of 5,6-EP (5,6α-EP plus 5,6β-EP) (0.44 mg) was present prior to heating, rose to 3.2 mg in 10 min and 17.7 mg in 30 min. However, with quercetin treatment, a level of 0.46 mg 5,6-EP (based on 100 g cholesterol) was formed initially, followed by an increment to 1.00 mg in 10 min and 2.83 mg in 30 min. This outcome is different from that reported by Chien *et al*. [17], as the inhibition of 7-OH (7α-OH plus 7β-OH), 7-keto and triol were not possible in the presence of 0.002% quercetin. Obviously a higher level of 0.02% quercetin employed in the present experiment should account for this effect.

Table 1 shows the total COPs formed during heating with or without quercetin at 150 °C. The amount of COPs generated during heating followed a time-dependent response for both treatments, with a lesser content for the treatment with quercetin. For instance, the amount of COPs produced with quercetin was 11.6-fold lower than that without quercetin after 30-min heating, demonstrating the potential of quercetin to be a powerful antioxidant.

Table 2 shows the percentage changes of residual cholesterol during thermal degradation at 150 °C. As it is difficult to estimate the concentration change of thermal degraded data (C_T_), the data in Table 2 were recalculated by combining the amounts of COPs (C_O_) and residual cholesterol (C_R_) at each interval, *i.e.*, total cholesterol = C_T_ + C_O_ + C_R_ or total cholesterol − C_T_ = C_O_ + C_R_ = actual residual cholesterol due to thermal degradation, so that the overall thermal degradation rate constant could be estimated. The residual concentrations of cholesterol were significantly different between treatments with and without quercetin for the same heating time. Nevertheless, a slight difference in cholesterol loss did occur after extensive heating for 60 and 90 min, as evidenced by a decline of 35.8 and 33.6% without quercetin respectively, in contrast to 19.5 and 22.9% with quercetin. This outcome implied that the thermal degradation of cholesterol proceeded fast in the beginning and a plateau was reached after prolonged heating. By comparing the data in Tables 1 and 2, a reversed trend was shown to occur for oxidative degradation, *i.e.*, it proceeded slower in the initial period and faster after extensive heating.

### 2.3. Kinetic Studies of Quercetin Degradation and Oxidation as well as Cholesterol Hydroperoxide Formation

The free radical chain reaction of cholesterol oxidation can be inhibited in the presence of quercetin, a free radical scavenger [5,6,19,20]. The initial oxidation and thermal degradation pathways of cholesterol as well as the thermal degradation and oxidation pathways of quercetin are shown in Figure 5. According to the kinetic studies of antioxidants by Pryor *et al*. [19] and Foti *et al*. [21], with quercetin the hydroperoxide formation rate can be expressed as follows:

(1) $$\frac{\text{d}[\text{COOH}]}{\text{d}t}=\frac{k[RH]R_{i}}{nk^{\prime }[Q]}$$

where [Q]: percentage concentration of quercetin

[COOH]: the concentration of hydroperoxide

[RH]: the concentration of target compound to be oxidized

R_i_: the initial reaction rate

k, k′: the reaction rate constant

n: number of free radicals scavenged per quercetin molecule

If all the thermal degradation, oxidation and free radical chain reactions for quercetin followed a first-order, the depletion rate of quercetin can be written by setting n. k′= k_f_ as follows:

(2) $$-\frac{\text{d}[\text{Q}]}{\text{dt}}={\text{k}}_{\text{d}}[\text{Q}]+{\text{k}}_{\text{O}}[\text{Q}]+{\text{k}}_{\text{f}}[\text{Q}]=({\text{k}}_{\text{d}}+{\text{k}}_{\text{O}}+{\text{k}}_{\text{f}})[\text{Q}]=k_{i}[Q]$$

where k_d_, k_o_, k_f_, and k_i_: the reaction rate constants (h^−1^) for thermal degradation, oxidation, free radical scavenging and the overall degradation, respectively.

According to the studies by Ozilgen and Ozilgen [22] and Chien *et al*. [12], the rate equation for forward formation of 7-OOH from cholesterol through free radical chain reaction (A→A′) is as follows:

(3) $$\frac{\text{d}[{\text{A}}^{\prime }]}{\text{dt}}={\text{k}}_{1}\left(1-\frac{\left[{\text{A}}^{\prime }\right]}{{\left[{\text{A}}^{\prime }\right]}_{\text{max}}}\right)\;[{\text{A}}^{\prime }]$$

where [A′]: percentage concentration of 7-OOH

[A′]_max_: the maximum attainable concentration of 7-OOH prior to degradation

k_1_: the reaction rate constant and t: time.

Combining Equations (1)–(3), the Equation (4) for 7-OOH formation could be obtained by setting [COOH] = [A′] as follows:

(4) $$\frac{\text{d}[{\text{A}}^{\prime }]}{\text{dt}}=\frac{{\text{k}}_{1}[{\text{A}}^{\prime }]}{{\text{k}}_{\text{f}}[\text{Q}]}\left[1-\frac{\left[{\text{A}}^{\prime }\right]}{{\left[{\text{A}}^{\prime }\right]}_{\text{max}}}\right]$$

For the second-order formation of 5,6-EP from cholesterol, the concentrations of both cholesterol and 7-OOH should be considered [12,23]. Thus, if we also consider the involvement of free radical scavenging effect of quercetin in epoxidation, the rate equations for 5,6-EP formation should be expressed as follows:

(5) $$\frac{\text{d}[\text{E}]}{\text{dt}}=\frac{{\text{k}}_{4}[\text{A}][{\text{A}}^{\prime }]}{{\text{k}}_{\text{f}}[\text{Q}]}$$

Furthermore, it was reported that thermal degradation of cholesterol follows first-order reaction [12,13] and the corresponding equation can be given as in Equation (1) in a previous study [17]. The integral form is as follows:

(6) $$[A]=[A_{0}]\;e^{-k_{5}t}$$

The following Equation (7) can be obtained by integrating of Equation (2).

(7) $$[\text{Q}]={\left[\text{Q}\right]}_{0}{\text{e}}^{-{\text{k}}_{\text{i}}\text{t}}$$

Likewise, Equations (7-1) and (7-2) were used for estimating quercetin degradation under nitrogen and oxygen, respectively. Both equations can be obtained by integration of the first-order rate equation of thermal degradation and oxidative degradation of quercetin as follows:

(7-1) $$[\text{Q}]={\left[\text{Q}\right]}_{0}{\text{e}}^{-{\text{k}}_{\text{d}}\text{t}}$$

(7-2) $$[\text{Q}]={\left[\text{Q}\right]}_{0}{\text{e}}^{-({\text{k}}_{\text{d}}+{\text{k}}_{\text{O}})\text{t}}$$

Integration after substituting Equations (7) into Equation (4) gave Equation (8) as follows:

(8) $$[{\text{A}}^{\prime }]=\frac{{\left[{\text{A}}^{\prime }\right]}_{\text{max}}}{1-\left(1-\frac{{\left[{\text{A}}^{\prime }\right]}_{\text{max}}}{{\left[{\text{A}}^{\prime }\right]}_{0}}\right)\omega }$$

where $\omega ={\text{e}}^{\frac{{\text{k}}_{1}}{{\left[\text{Q}\right]}_{0}\;{\text{k}}_{\text{p}}}\left(1-{\text{e}}^{{\text{k}}_{\text{i}}\text{t}}\right)}$

k_P_ = k_i_ × k_f_

[A]_0_,[A′]_0_,[E]_0_,[Q]_0_: concentration of cholesterol, 7-OOH, 5,6-EP and quercetin at 0-min heating

Integration after substitution of Equations (6), (7) and (8) into Equation (5), the following Equation (9) can be obtained.

(9) $$[\text{E}]={\left[\text{E}\right]}_{0}+\frac{{\text{k}}_{4}{\left[\text{A}\right]}_{0}{\left[{\text{A}}^{\prime }\right]}_{\text{max}}}{{\text{k}}_{\text{p}}{\left[\text{Q}\right]}_{0}}\int_{0}^{\text{t}}\frac{{\text{e}}^{(k_{i}-k_{5})\text{t}}}{1-\left(1-\frac{{\left[{\text{A}}^{\prime }\right]}_{\text{max}}}{{\left[{\text{A}}^{\prime }\right]}_{0}}\right)\omega }\text{dt}$$

Among the above equations, Equations (6), (7) and (8) are in the forms of exact integration. As the exact integrations for Equation (9) are not possible, we can only get a better approximation to the integral term in equation by using a quadratic polynomial of Taylor’s series as follows:

(10) $$\begin{array}{l} {}[\text{E}]={\left[\text{E}\right]}_{0}+\frac{{\text{k}}_{4}{\left[\text{A}\right]}_{0}{\left[{\text{A}}^{\prime }\right]}_{0}}{{\text{k}}_{\text{p}}{\left[\text{Q}\right]}_{0}({\text{k}}_{\text{i}}-{\text{k}}_{5})}[(1+{\text{m}}_{3}(1-{\text{m}}_{2})\text{t}+{\text{m}}_{4}{\text{t}}^{2}){\text{e}}^{(k_{i}-k_{5})\text{t}}-\left(\frac{{\text{m}}_{3}(1-{\text{m}}_{2})+2{\text{m}}_{4}\text{t}}{{\text{k}}_{\text{i}}-{\text{k}}_{5}}\right){\text{e}}^{(k_{i}-k_{5})\text{t}}\\ +\frac{2{\text{m}}_{4}}{{\left({\text{k}}_{\text{i}}-{\text{k}}_{5}\right)}^{2}}{\text{e}}^{(k_{i}-k_{5})\text{t}}-1+\left(\frac{{\text{m}}_{3}(1-{\text{m}}_{2})}{{\text{k}}_{\text{i}}-{\text{k}}_{5}}\right)-\frac{2{\text{m}}_{4}}{{\left({\text{k}}_{\text{i}}-{\text{k}}_{5}\right)}^{2}}] \end{array}$$

where m_2_ = [A′]_0_/A′]_max_;

m_3_ = k_1_/k_f_[Q]_0_;

m_4_ = m_3_(2m_3_m_2_^2^ - m_2_k_i_ - 3m_2_m_3_ + k_i_ + m_3_).

The experimental data were thus analyzed in sequence and fitted best to the corresponding non-linear regression model. The residual percentage of quercetin in Figure 4 was used for computing the reaction rate constants (h^−1^) for thermal degradation (k_d_), oxidation (k_o_) and the overall degradation (k_i_). The Equations (7-1), (7-2) and (7) implied that for a first-order reaction, the natural logarithm of the residual quercetin percentage is a linear function of time and thus, k_d_, k_o_, and k_i_ in Equations (7-1), (7-2) and (7) can be estimated using a linear regression model [24]. The results are shown in Table 3, with K_d_ being 0.253 h^−1^ and correlation coefficients (r^2^) 0.94, which was lower than that for oxidative degradation of quercetin (k_O_ (h^−1^) = 0.615 = 0.868 - 0.253). Although the correct value of k_f_ for quercetin degradation in the free radical chain reaction should be evaluated from Equation (8), the k_f_ (h^−1^) values from Equations (7-2) and (7) (6.302 h^−1^ or 7.17 - 0.868 h^−1^) can still be used for subsequent comparison, and was approximately 24.9 and 10.2-folds higher than k_d_ or k_O_, respectively.

The data in Table 2 were used for computing k_5_ in Equation (6) by using a linear regression model [24], with K_5_ (h^−1^) being 0.19 ± 0.01 (r^2^ = 0.99), which was lower than the treatment without quercetin (0.94 ± 0.03) by 4.9-fold (Table 4). The rate constants in Equations (8) and (10) were estimated by using the least squares method with a nonlinear Marquardt iterative method until the convergences of best-fitted parameters were met. Table 4 shows the rate constants of the reaction pathways of cholesterol peroxidation and epoxidation as well as free radical chain reaction and degradation of quercetin over a 30-min heating period. With quercetin, the r^2^ of all the reactions were higher than 0.82, especially for the free radical chain reaction of quercetin, as a high r^2^ (0.94) was found after a non-linear estimation from Equation (8) with a k_f_ (h^−1^) of 3.28. This value was close to that estimated from Equation (7), *i.e.*, 6.302 = 7.17 - 0.868 in Table 3 by a difference of 1.9-fold. Though we do not have a plausible explanation for the difference in k_f_ value, it may be possibly due to the low amount of COPs formed over a 30-min heating period (Table 1), which made it more difficult to estimate the k_f_ value from Equation (8). By combining all the results shown above, it is apparent that all the reactions involved for quercetin during heating, including thermal degradation, oxidative degradation and free radical chain reaction, fit well the first-order model. In addition, the formation of 7-OOH, the initial oxidation product generated from peroxidation of cholesterol, was further plotted based on the corresponding rate equation, *i.e.*, Equation (8) (Figure 7) and the rate constants are listed in Table 4. Both curves fitted well with the data points with or without quercetin. All these findings suggested that the Equation (8) could successfully estimate the degradation of cholesterol and quercetin over a 30-min heating period.

For cholesterol oxidation in the presence of quercetin, the r^2^ of peroxidation and epoxidation were 0.94 and 0.82, respectively, whereas the free radical chain reaction of quercetin was 0.94 (Table 4). It may be postulated that both formation of 7-OOH and 5,6-epoxide during heating of cholesterol with quercetin fit the second-order, but the free radical chain reaction of quercetin fits the first-order.

It was also observed that the k_1_ (h^−1^) dropped sharply from 488.2 for the treatment without quercetin to 1.8 × 10^−4^ for the treatment with quercetin, implying the incorporation of 0.02% quercetin to be effective in inhibiting free radical formation. Similarly, a substantial decline in k_4_ by 2.7 × 10^5^-fold after quercetin treatment was shown for epoxidation, which may be accounted for by a pronounced inhibition of 7-OOH formation during heating with quercetin. By comparison, the rate constants (h^−1^) of the major reaction pathways of cholesterol oxidation in Table 4 followed the order: k_5_ >> k_4_ > k_1_ for the quercetin treatment, and k_4_ > k_1_ >> k_5_ for the treatment without quercetin.

## 3. Experimental Section

### 3.1. Materials

Several COPs standards, including 7β-hydroxycholesterol (7β-OH), 7α-hydroxycholesterol (7α-OH), 5,6α-epoxycholesterol, (5,6α-EP), 5,6β-epoxycholesterol, (5,6β-EP), 7-ketocholesterol (7-keto) and 5α-cholestane-3β,5,6β-triol (triol) were purchased from Sigma (St. Louis, MO, USA) and Steraloids Inc. (Wilton, NH, USA). Standards of cholesterol, quercetin and lauryl alcohol were also from Sigma and were used without further purification. The HPLC-grade solvents such as n-hexane, ethanol, methanol and isopropanol were from Mallinckrodt Co. (Paris, KY, USA). The analytical grade solvent 1,2-dichloroethane was from Riedel-de Haën Co. (Barcelona, Spain). Paraffin oil, acetonitrile, phosphoric acid and potassium dihydrogen phosphate were from Merck (Darmstadt, Germany). The NH_2_ cartridges were from Unichrom Scientific (Taipei, Taiwan). The Strata C18-E solid phase extraction cartridges (500 mg/3 mL, 55 μm, 70 Å) were from Phenomenex Co. (Torrance, CA, USA).

### 3.2. Instrumentation

The HPLC instrument was composed of a Jasco PU980 pump (Jasco Co., Tokyo, Japan), an UV-VIS detector, a Jasco 830 refractive index detector and an Agilent 1100 series G1316A column temperature controller (Palo Alto, CA). A Borwin software system was used to process data. A Vydac 201TP54 C18 column (250 × 4.6 mm i.d., 5-μm particle size; Vydac Inc., Hesperia, CA, USA) was used to separate and quantify quercetin in heated samples. Two Merck Lichrospher 100 CN columns (244 × 2.4 mm i.d.) with both containing 5-μm packing material were used to separate and quantify cholesterol as well as COPs in heated samples. The Sorvall RC5C high-speed centrifuge was from Du Pont (Wilmington, DL).

### 3.3. Heating of Quercetin with or without Cholesterol

A mixture containing 0.6-mL of lauryl alcohol and 2.4-mL of paraffin oil was poured into a 100-mL round-bottom flask, followed by adding 600 mg of cholesterol standard and 0.12 mg (0.02% of cholesterol) of quercetin dissolved in 2-mL of ethanol. Lauryl alcohol was used as a solvent to dissolve the mixture of cholesterol and quercetin, as well as induce triol formation in an aqueous system through alcoholysis of the epoxy-containing COPs [17]. The flask was soaked in a paraffin-oil bath, which was preheated for 5 min with nitrogen gas flushing into the flask at the same time until the internal temperature reached 150 ± 1 °C. Then, oxygen was pumped into the flask during heating for 0, 5, 10, 20 and 30 min. After heating, the flask was inserted into dry ice to terminate the reaction and then each sample was analyzed by HPLC. For the other two treatments, the same procedure as described for heating of quercetin with cholesterol was used with the exception that 1.0 mg of quercetin was heated alone and the heating times were changed to 0, 5, 10, 30, 60, 90 and 120 min, with nitrogen or oxygen being pumped-in separately. For each heating treatment, triplicate experiments were carried out and the data were analyzed by a non-linear regression method [24].

### 3.4. Extraction and Purification of Cholesterol and COPs

A method based on Nourooz-Zadeh [25] was modified for extraction and purification of COPs from the samples containing quercetin and cholesterol. Each heated sample was mixed with 20-mL of hexane-isopropanol (3:2, v/v), and the solution was flushed with nitrogen gas and shaken vigorously for 3 min in a sealed flask. Then the mixture was centrifuged at 26,000 × g for 5 min at 25 °C. The upper layer was collected, transferred to a centrifuge tube and 12-mL of distilled water was added, after which the mixture was centrifuged again for another 5 min, followed by collecting the upper phase and evaporating to dryness at 35 °C under vacuum. The residue was dissolved in 1-mL of hexane-1,2-dichloroethane (1:1, v/v) and the solution was poured into a NH_2_ cartridge for purification. Initially, 5-mL of hexane was added to the cartridge to remove impurities such as paraffin oil, followed by adding 25-mL of hexane-1,2-dichloroethane-isopropanol (50:30:15, v/v/v) to elute cholesterol and COPs. The eluate was evaporated to dryness at 35 °C under vacuum, with the residue dissolved in hexane-isopropanol (95:5, v/v) and filtered through a 0.2-μm membrane filter for HPLC analysis.

### 3.5. Extraction and Purification of Quercetin

Each heated quercetin sample containing cholesterol was mixed with 7.5-mL of hexane-isopropanol (3:2, v/v) in a flask, the mixture was then flushed with nitrogen gas and shaken vigorously for 3 min, after which 3-mL of distilled water was added and the mixture was shaken vigorously for another 3 min. Then the solution was centrifuged at 26,000 × g for 5 min at 25°C, followed by collecting the lower layer containing quercetin, pouring into a centrifuge tube, adding 3-mL of absolute ethanol, centrifuging again for 5 min and collecting the lower phase. Next, a 500-μL of the lower phase was poured into a C18-E solid phase extraction cartridge, which was previously activated with 3-mL of methanol. Five mL of water-acetonitrile (1:1, v/v) was added to elute quercetin, and the elute was filtered through a 0.2-μm membrane filter for HPLC analysis.

### 3.6. HPLC Analysis of Quercetin

The heated sample containing quercetin was dissolved in 20-mL of acetonitrile/phosphate buffer (pH 4) (55:45, v/v). Six concentrations of 1, 5, 10, 15, 20 and 25 mg/L of quercetin were each prepared in the same buffer solution. All the quercetin standard solutions were filtered through a 0.2-μm membrane filter and a 20-μL sample was injected into HPLC. A mobile phase of acetonitrile-phosphoric acid buffer (pH 4) (55:45, v/v) and a Vydac 201TP54 C18 column with flow rate at 1.5 mL/min was used to separate and quantify quercetin at 460 nm. The standard curve of quercetin was obtained by plotting concentration against peak area, and the regression equation and correlation coefficient (r^2^) were calculated. A high recovery of 98% was obtained when quercetin standard was added and subjected to the same extraction and purification procedure. Triplicate analyses were conducted and the mean values were determined.

### 3.7. TLC Analysis of COPs

The Wurster dye was prepared as described by Smith and Hill [18]. One g of *N*,*N*-dimethyl-*p-*phenylenediamine dihydrochloride was dissolved in 100 mL of 50% methanol solution, followed by shaking the mixture thoroughly, adding 1-mL of glacial acetic acid, pouring the solution into a glass vial and storing at −20 °C until use. For TLC analysis, the plate (20 × 20 cm) precoated with silica gel with a thickness of 250 μm was lined with a filter paper in a developing glass tank. A mobile phase of 200 mL of benzene-ethyl acetate (60:40, v/v) was poured into the tank and allowed to equilibrate for 30 min for vapor saturation prior to development [26]. A micropipette was used to spot a 10-μL volume of extract onto the glass plate, and the chromatogram was developed for a distance of 18 cm at 25 °C, after which the plate was dried in an oven at 110 °C for 10 min, followed by spraying with 50% H_2_SO_4_. The color development of COPs under UV radiation at 254 nm was observed and recorded. In addition, the red spot was further subjected to HPLC analysis.

### 3.8. HPLC Analysis of COPs

Two LiChrospher 100 CN columns connected in series and an isocratic solvent system of hexaneisopropanol (95:5, v/v) with flow rate at 1.0 mL/min were used to separate cholesterol and various COPs with RI detection. Identification was accomplished by comparing retention times of unknown peaks with authentic standards and cochromatography with added standards. An external calibration method was employed for quantification of various COPs. Seven concentrations of cholesterol (0.5, 1.0, 2.5, 5, 10, 25 and 50 mg/mL) and COPs (50, 100, 200, 500, 1000, 2500 and 5000 μg/mL) were each prepared and the standard curve was obtained by plotting concentration against area after injecting into HPLC. The regression equations and correlation coefficients (r^2^) were obtained by using a Borwin software system. Recovery was carried out by adding a fixed concentration of cholesterol and various COPs standards to samples for extraction and HPLC analysis, and a high recovery ranging from 97–100% was attained.

### 3.9. Kinetic Analysis of COPs and Quercetin

The concentration changes of quercetin during heating in the presence of nitrogen, oxygen or cholesterol were statistically analyzed using a non-linear regression procedure of SAS [24]. Also, the formation of COPs and degradation of cholesterol were analyzed statistically. The rate constant of each reaction was determined based on the least square method, *i.e.*, the Marquardt iterative procedure, until the convergence of all the parameters was best fitted. The precision of the parameters for each kinetic equation was also assessed. Analysis of variance and comparison of the residual amounts of cholesterol at various heating time intervals were carried out by using the SAS system [24]. After a preliminary F test, the differences among the residual cholesterol were analyzed by Duncan’s multiple range test (p < 0.05).

## 4. Conclusions

In conclusion, the major reaction pathways during heating of quercetin with and without cholesterol at 150 °C included cholesterol peroxidation and epoxidation, as well as thermal degradation, oxidative degradation and free radical chain reaction of quercetin. The correlation coefficients (r^2^) for all the reactions ranged from 0.82–0.99 based on non-linear regression analysis for cholesterol when heated with quercetin. The reactions for 7-OOH and 5,6-epoxide formation fitted the second-order, while all the reaction for quercetin degradation and free radical reaction fitted the first-order. The kinetic model developed in this study may be applied to predict the concentration changes of quercetin degradation in the presence and absence of cholesterol during heating at 150 °C.

## Figures and Tables

**Table 1:** Inhibitory effect of quercetin on total COPs formed during heating at 150 °C.

Heating time (min)	COPs (%)^a^	
	Control^b^	Quercetin^b^
0	0.08 ± 0.00 ^A^,^a^	0.07 ± 0.00 ^A^,^b^
5	0.18 ± 0.01 ^A^,^a^	0.09 ± 0.00 ^A^,^b^
10	0.94 ± 0.01 ^B^,^a^	0.16 ± 0.01 ^A^,^b^
30	5.94 ± 0.05 ^C^,^a^	0.51 ± 0.00 ^B^,^b^
60	15.4 ± 0.3^D^,^a^	2.05 ± 0.03 ^C^,^b^
90	30.9 ± 0.5^E^,^a^	7.77 ± 0.06 ^D^,^b^
120	—c	24.4 ± 0.4 ^E^

a Values are expressed as percentage relative to cholesterol content at 0 min heating time; mean ± standard deviation of triplicate determinations.

b Symbols bearing the letters A-E in the same column or a-b in the same row are significantly different (p < 0.05);

c Not detected.

**Table 2:** Percentage changes of residual cholesterol due to thermal degradation at 150 °C.

Heating time (min)	Cholesterol (%) a	
	Control b	Quercetin b
0	97.9 ± 0.6 ^A^,^a^	96.9 ± 0.3 ^A^,^a^
5	93.2 ± 1.05 ^B^,^a^	95.7 ± 0.2 ^A^,^b^
10	87.5 ± 0.4 ^C^,^a^	94.1 ± 0.1 ^A^,^b^
30	69.0 ± 1.0 ^D^,^a^	86.4 ± 0.1 ^B^,^b^
60	64.2 ± 0.9 ^E^,^a^	80.5 ± 0.3 ^C^,^b^
90	66.4 ± 0.7 ^E^,^a^	77.1 ± 0.5 ^D^,^b^
120	—c	77.4 ± 2.4 ^D^

a Values are expressed as percentage relative to cholesterol content at 0 min heating time; mean ± standard deviation of triplicate determinations.

b Symbols bearing different letters of A-E in the same column or a-b in the same row are significantly different (p < 0.05);

c Not detected.

**Table 3:** Oxidation and degradation rate constants of quercetin during heating at 150 °C.

Treatment	Rate equations a	k (h^−1^)	r^2^
Nitrogen	$\text{Q}\overset{\text{kd}}{\to }{\text{Q}}_{\text{d}}$	0.253 ± 0.027	0.94
Oxygen	$\text{Q}\overset{\text{kd}+\text{ko}}{\to }{\text{Q}}_{\text{d}}+{\text{Q}}_{\text{o}}$	0.868 ± 0.019	0.99
Oxygen and Cholesterol	$\text{Q}\overset{\text{kd}+\text{ko}+\text{kf}}{\to }{\text{Q}}_{\text{d}}+{\text{Q}}_{\text{o}}+{\text{Q}}^{•}$	7.17 ± 0.67	0.91

a Q (quercetin), Q_d_ (degraded products of quercetin), Q_o_ (oxidized products of quercetin), Q^•^ (quercetin free radical) and k_d_, k_o_ and k_f_ are the rate constants.

**Table 4:** Oxidation and degradation rate constants of cholesterol during heating at 150 °C.

Rate equations a	Reactions	C b		Qu b	
		k (h^−1^)	r^2^	k (h^−1^)	r^2^
$\text{Chol}\overset{\text{k}1}{\to }7-\text{OOH}$	Free radical chain	488.2 ± 0.2	1.00	1.8×10^−4^ ± 0.1×10^−4^	0.94
$\text{Chol}\overset{\text{k}4}{\to }5,6-\text{EP}$	Epoxidation	4240.8 ± 344.7	0.89	0.016 ± 0.001	0.82
$\text{Chol}\overset{\text{k}5}{\to }\text{D}$	Degradation	0.94 ± 0.03	0.99	0.19 ± 0.01	0.99
$\text{Q}\overset{\text{kf}}{\to }{\text{Q}}^{•}$	Free radical chain	—c	—	3.28 ± 0.00	0.94

a Chol (cholesterol), 7-OOH (7-hydroperoxycholesterol), 5,6-EP (5,6-epoxycholesterol), D (degraded product of cholesterol), Q (quercetin), Q^•^ (quercetin free radical) and k_1_, k_4_, k_5_ and k_f_ are the rate constants.

b C: autoxidation of cholesterol without quercetin. Qu: the initial stage during heating of cholesterol with quercetin (t ≤ 30 min).

c No detected reaction product.
